# Supplementary material for: Effects of Long-Term Dietary Protein Restriction on Intestinal Morphology, Digestive Enzymes, Gut Hormones, and Colonic Microbiota in Pigs
Source: Animals (Basel). 2019 Apr 20;9(4):180. doi: 10.3390/ani9040180 (PMC6523122; doi:10.3390/ani9040180)
Supplement: Supplementary file 1 [file animals-09-00180-s001.pdf]

animals

MDPI

*Article*

# Effects of Long-Term Dietary Protein Restriction on Intestinal Morphology, Digestive Enzymes, Gut Hormones, and Colonic Microbiota in Pigs

**Defu Yu, Weiyun Zhu and Suqin Hang**

**Supplementary Materials:**

**Table S1.** Primers used for bacterial DNA amplification for microbiota composition analysis.

| Target Organisms | Primers [1] | Sequences (5′-3′) | Annealing Temperature (°C) |
|---|---|---|---|
| Total bacteria | Total-F<br>Total-R | GTGSTGCAYGGYYGTCGTCA<br>ACGTCRTCCMCNCCTTCCTC | 60 |
| *Bacterodietes* | Bac 964-F<br>Bac 1060-R | GGARCATGTGGTTTAATTCGATGAT<br>AGCTGACGACAACCATGCAG | 60 |
| *Lactobacillus* | Lac-F<br>Lac-R | AGCAGTAGGGAATCTTCCA<br>ATTCCACCGCTACACATG | 61 |
| *Enterobacteriaceae* | Eco-F<br>Eco-R | CATGCCGCGTGTATGAAGAA<br>CGGGTAACGTCAATGAGCAAA | 59 |
| *Clostridium cluster XIV* | Clo14-F<br>Clo14-R | CGGTACCTGACTAAGAAGC<br>AGTTTYATTCTTGCGAACG | 60 |
| *Clostridium cluster IV* | C. leptum-F<br>C. leptum-R | GCACAAGCAGTGGAGT<br>CTTCCTCCGTTTTGTCAA | 60 |
| *Ruminococcus* | Rum-F<br>Rum-R | CGAACGGAGATAATTTGAGTTTACTTAGG<br>CGGTCTCTGTATGTTATGAGGTATTA CC | 60 |

[1] F: forward primer; R: reverse primer.

**Table S2.** Richness and diversity estimator of microbiota in the colonic luminal contents of pigs.

| | NP | MP | LP | SEM | *p*-Value |
|---|---|---|---|---|---|
| Ace | 423.48 | 422.91 | 365.44 | 34.363 | 0.633 |
| Chao | 427.92 | 431.93 | 367.67 | 25.114 | 0.673 |
| Shannon | 3.102 | 3.171 | 2.946 | 0.191 | 0.395 |
| Simpson | 0.141 | 0.142 | 0.171 | 0.018 | 0.795 |
| Coverage | 0.998 | 0.998 | 0.998 | <0.001 | 0.503 |

NP: normal protein diets according to the National Research Council (NRC, 2012); MP: reduced protein by 3% compared to the NP diet supplemented with Lys, Met, Thr, and Trp; LP: reduced protein by 6% compared to the NP diet supplemented with Lys, Met, Thr, and Trp. SEM: standard error of the mean. [a,b,c] Mean values in the same row differ in significance ($p < 0.05$).
